# Laser microdissection system based on structured light modulation dual cutting mode and negative pressure adsorption collection

**DOI:** 10.1371/journal.pone.0308662

**Published:** 2024-08-26

**Authors:** Bocong Zhou, Caihong Huang, Dingrong Yi

**Affiliations:** 1 College of Mechatronics and Automation, Huaqiao University, Xiamen, China; 2 College of Mechanical Information Science and Engineering, Huaqiao University, Xiamen, China; University of Science and Technology of China, CHINA

## Abstract

Laser microdissection technology is favored by biomedical researchers for its ability to rapidly and accurately isolate target cells and tissues. However, the precision cutting capabilities of existing laser microdissection systems are hindered by limitations in overall mechanical movement accuracy, resulting in suboptimal cutting quality. Additionally, the use of current laser microdissection systems for target acquisition may lead to tissue burns and reduced acquisition rates due to inherent flaws in the capture methods. To address these challenges and achieve precise and efficient separation and capture of cellular tissues, we integrated a digital micromirror device (DMD) into the existing system optics to modulate spatial light. This allows the system to not only implement the traditional point scanning cutting method but also utilize the projection cutting method.We have successfully cut various patterns on commonly used laser microdissection materials such as PET films and mouse tissues. Under projection cutting mode, we were able to achieve precise cutting of special shapes with a diameter of 7.5 micrometers in a single pass, which improved cutting precision and efficiency. Furthermore, we employed a negative pressure adsorption method to efficiently collect target substances. This approach not only resulted in a single-pass capture rate exceeding 90% for targets of different sizes but also enabled simultaneous capture of multiple targets, overcoming the limitations of traditional single-target capture and enhancing target capture efficiency, and avoiding potential tissue damage from lasers.In summary, the integration of the digital micromirror device into laser microdissection systems significantly enhances cutting precision and efficiency, overcoming limitations of traditional systems. This advancement demonstrates the accuracy and effectiveness of laser microdissection systems in isolating and capturing biological tissues, highlighting their potential in medical applications.

## 1. Introduction

Cells, which are the fundamental units of biological processes, are essential for biomedical research [1–3]. However, it is important to acknowledge that cells do not exist in isolation. The presence of different cell types in a mixture often obscures the true nature of a lesion. As a result, it is now a major challenge for biological researchers to isolate single cells from specific anatomical regions in complex heterogeneous tissues. To address this problem, scientists have conducted extensive research that has led to the development of various cell separation methods [4–6]. Laser microdissection is a commonly used technique for non-contact method for microdissection and isolation of biological samples under a microscope. When laser microdissection is conducted, the targeted material absorbs UV laser energy, converting it into internal heat energy. As the material’s temperature rises, it eventually reaches a critical point, leading to vaporization on the material’s surface. The vaporized material is then expelled from the processing area, creating a precise cutting opening. Laser microdissection technology enables extremely fine cutting with smooth edges, free from burrs, and does not produce carbonization. This technique ensures sample purity and high precision, making it invaluable for research in molecular biology, cytogenetics, and related fields [7–10]. In a study by Laura W. Harris et al. [11], laser microdissection was utilized to isolate microvascular endothelial cells and neurons from postmortem brain tissues of both schizophrenic patients and healthy individuals. The objective of their investigation was to elucidate microvascular system dysfunction in schizophrenia patients. Similarly, Eulalie Buffin et al. [12] employed laser microdissection to procure Drosophila precursor cells, aiming to study the mechanisms involved in their specification. Another study conducted by Selda Aydin et al. [13] employed laser microdissection to procure lesions, facilitating the characterization of the TP53 mutation spectrum in malignant urothelial tissues of patients with aristolochic acid nephropathy in Belgium.

The laser microdissection system utilized in the aforementioned studies relied solely on mechanical motion for point-scanning cutting. Two prevalent approaches to point scanning cutting methods in laser microdissection systems include stage movement and galvanometer systems [14–16]. Nevertheless, the system’s limited mechanical movement accuracy may result in incomplete trajectories or low contour quality when cutting small targets. Consequently, it is imperative to develop a laser microdissection system that is both straightforward and capable of effectively isolating complex tissues. This advancement will significantly enhance the utility of laser microdissection technology.

A fully functional laser microdissection system must accurately isolate the desired tissue and capture anatomical targets with precision. Currently, various laser capture microdissection systems are available on the market, including those offered by prominent companies such as American Thermo Fisher, German Leica, German Zeiss, and German MMI [17–19]. These systems employ diverse collection methods. For instance, some systems utilize adhesion capture methods whereby infrared light illuminates a thermoplastic membrane placed on cells to facilitate cell adhesion and capture [20]. However, this method exposes target cells to temperatures of up to 90 degrees Celsius [21], potentially causing thermal damage and mechanical damage during the pulling process. Additionally, this collection method may pose contamination risks. Another approach is gravity-based collection, where the cut sample falls into a collection tube under the influence of gravity [22]. This method, however, is unsuitable for cell environments requiring culture media, and there is a risk of losing target cells due to an uncontrolled falling process. Moreover, there is a collection method employing a refocused ultraviolet cutting laser that emits laser pulses, with the pressure generated by these pulses ejecting the target into a container directly above. This method, however, may potentially damage cellular DNA and RNA. Therefore, there is an urgent need for a novel and more universal method to capture target tissues.

In order to tackle the aforementioned challenges, this paper proposes a laser microdissection system that utilizes digital micromirror device (DMD) technology. In this system, the incident laser beam is shaped by the DMD to accommodate complex cutting trajectories and target tissues of varying sizes. When dealing with small targets characterized by intricate curved trajectories, the laser beam can be precisely shaped to match the contour of the area to be cut. This enables one-time cutting without the need for mechanical movement, thereby enhancing both cutting accuracy and efficiency. The point-scanning cutting method remains suitable for cutting large volume targets or tissues with simple trajectories. Moreover, the device incorporates negative pressure to ensure stable capturing of targets of different sizes and can simultaneously capture multiple targets.

## 2. Materials and methods

### 2.1 Laser microdissection method based on DMD

**Fig 1:**
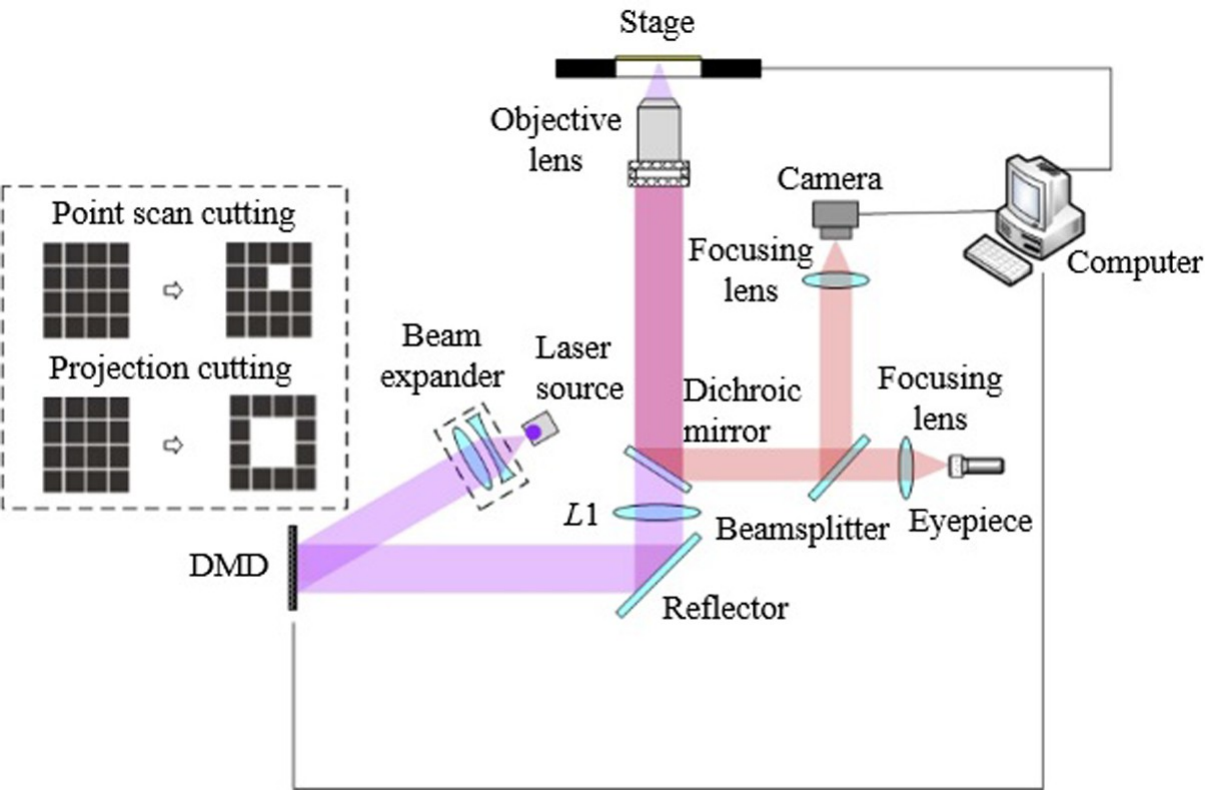
Schematic diagram of laser microdissection.

Fig 1 depicts the essential instruments and overall layout of the experimental platform for the laser micro-cutting system utilizing digital micromirror device (DMD) technology.

The laser microdissection system detailed in this article comprises multiple components, including a UV laser, a beam expander, a digital micromirror device (DMD), a zoom lens group, and a microscope system. The laser implemented is a diode-pumped Q-switched solid-state laser produced by Spectra-Physics, specifically the EONE-349-120 model, featuring a center wavelength of 349 nm. The microscope system showcases an Olympus IX73 inverted fluorescence microscope, with technical specifications elaborated in Table 1. The DMD device utilized is the DLP7001 from Texas Instruments, with comprehensive technical specifications outlined in Table 2. Throughout the laser microdissection procedures, the UV laser beam is directed towards the DMD through a laser beam expander. Subsequently, the DMD shapes the incident beam and projects it based on the preloaded pattern. The laser beam undergoes zooming via a telescope system before being focused onto the tissue surface.

**Table 1:** Main technical parameters of the ultraviolet laser.

Technical Parameter	Value
Wavelength/(nm)	349
Pulse Width/(ns)	<5@1 kHz
Average Power Output/(mw)	120@1 kHz
Repetition Rate/(KHZ)	Single shot to 5

**Table 2:** Main technical parameters of DMD.

Technical Parameter	Value
DMD micromirror array	1024×768
waveband	VIS,UV
Lens size/(μm)	13.68
Target size/(mm^2^)	14.00×10.50
RAM/(Gbits)	64/128
Refresh rate(1bit/8bit)/(HZ)	22727/290
PC transmission rate/(fps)	>4000

### 2.2 DMD laser beam shaping principle

**Fig 2:**
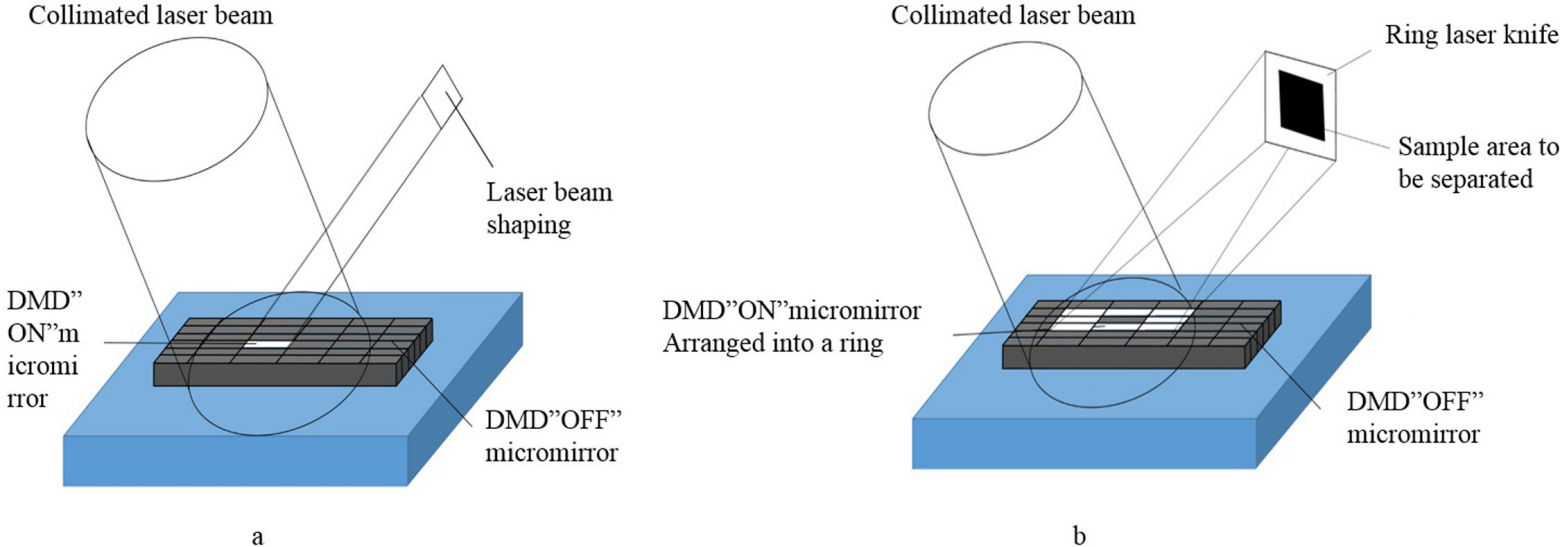
Principle of DMD beam shaping. (a) Point cutting mode. (b) Nonmechanical motion cutting mode.

To facilitate projection cutting, a diffractive spatial light modulator, specifically a digital micromirror device (DMD), was integrated into the system. The DMD offers several advantages over existing galvanometer systems in terms of spatial light modulation, including high-speed switching capabilities, high display resolution, precise phase control, and enhanced stability and reliability. Additionally, the DMD benefits from mass production and cost advantages. The principle of laser beam shaping in the DMD relies on controlling the state of its micromirror elements. By toggling the state of these elements, any desired projection shape can be achieved [23]. Fig 2 illustrates the fundamental process of DMD laser beam shaping. In panel (a), micromirrors are utilized to generate open "spots" with specific diameters. In panel (b), a closed-loop polygonal cutting trajectory can be attained by activating the target micromirror element while deactivating corresponding micromirror elements at other locations.

**Fig 3:**
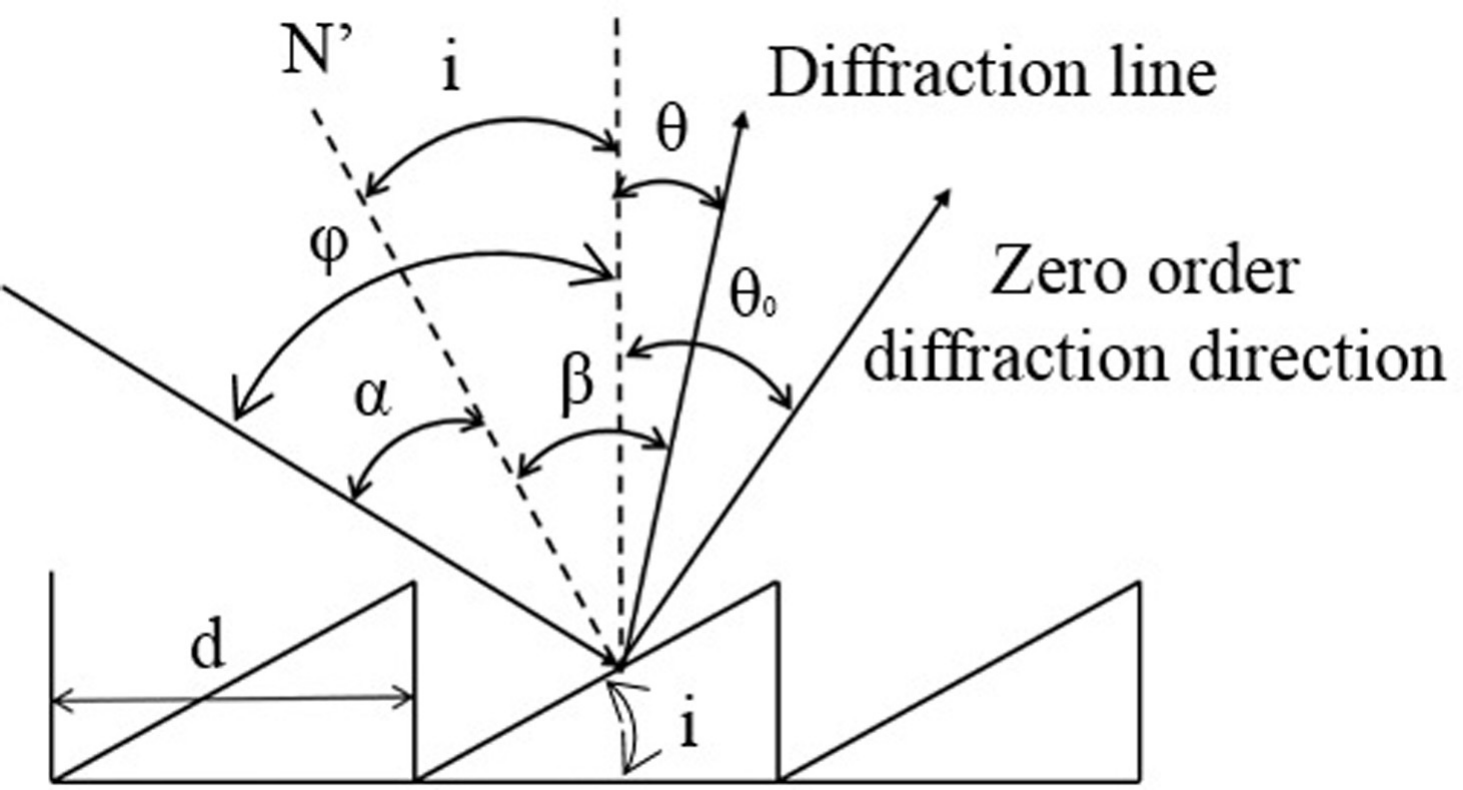
Schematic diagram of DMD blazed grating model.

**Fig 4:**
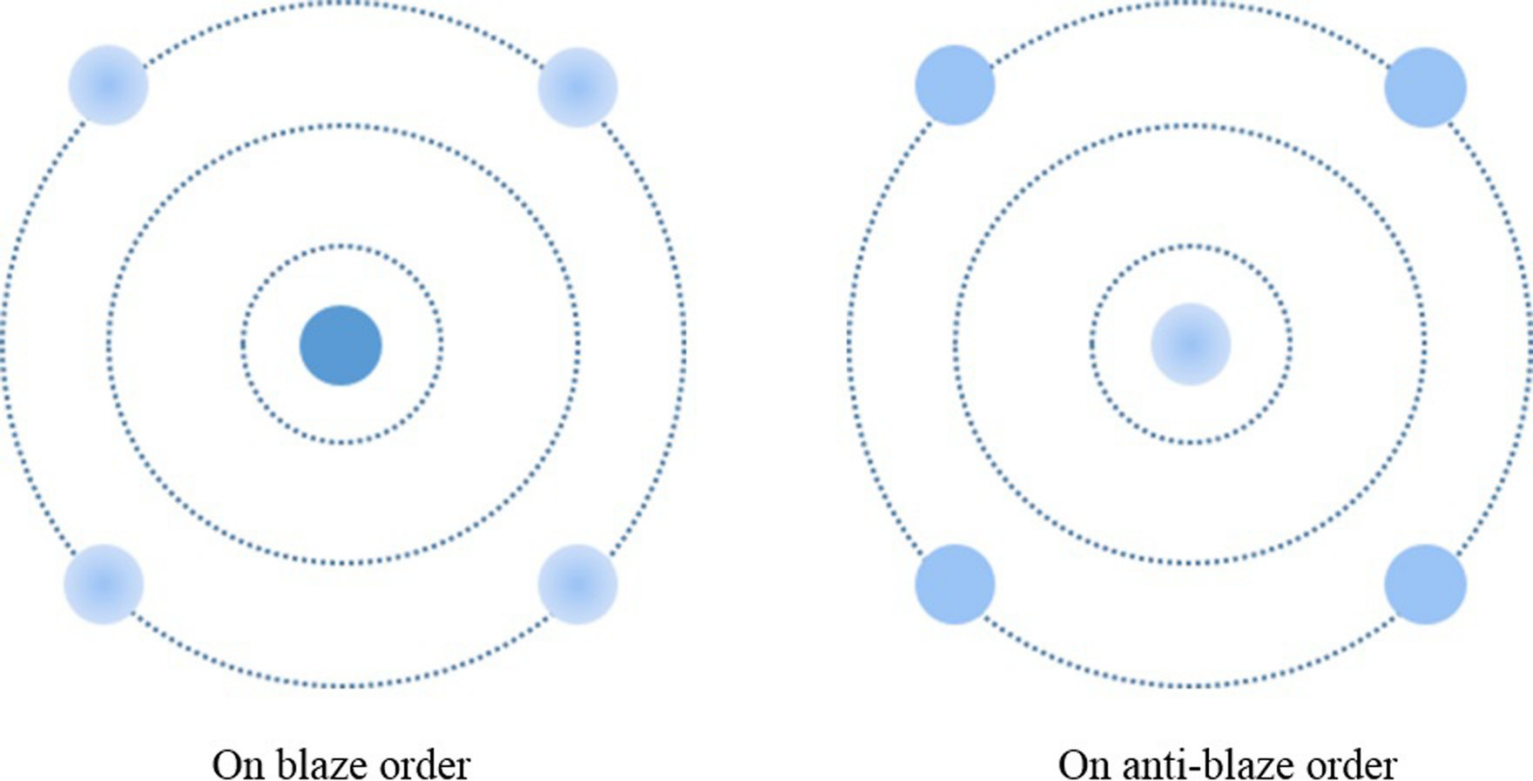
Diffraction orders with coherent illumination.

The reflection produced by the DMD yields a two-dimensional pattern of spots known as diffraction orders. Depending on factors such as the pixel pitch, the tilt angle of the DMD micromirrors, the wavelength of the illumination, and the angle of incidence of the illuminating light, there can be a spectrum ranging from fully blazed to fully anti-glare conditions. A blaze condition occurs when a single diffraction order contains the majority of the energy within the entire diffraction pattern, representing the optimal scenario. Conversely, an anti-blaze condition arises when the four brightest orders in the diffraction pattern possess equal amounts of energy, a situation to be avoided in this system [24–26]. The diffraction diagram of the DMD is depicted in Fig 3, while Fig 4 illustrates the energy distribution of DMD diffraction patterns in both blazed and non-blazed states. The color depth indicates the intensity of the energy within the diffraction orders.

Once the micromirror size and laser wavelength are determined, the relationship between the energy distribution of specific diffraction orders and the incident angle can be described using the diffraction model corresponding to the blazed grating model, as follows:

d(sinφ∓sinθ)=mλ (1)

The blaze angle, denoted as *i*, represents the angle formed between the groove surface and the grating surface. The grating constant *d*, signifies the distance separating two adjacent grooves. The incident angle *φ* indicates the angle formed between the incident light and the normal to the grating plane. Finally, the diffraction angle *θ*, denotes the angle between the diffracted light ray and the normal line of the grating plane.

Given that *a* = *φ*–*i* and *β* = *θ*+ *i*, substituting these expressions into Eq (1) results in:

2dsinicos(φ−i)=mλ (2)

This approach establishes the conditions necessary to achieve blaze for the mth diffraction order. The wavelength associated with the peak light intensity is termed the blaze wavelength. When the incident angle is equal to the blaze angle, implying that the incident light is perpendicular to the sawtooth surface of the blazed grating, with the incident angle *φ* = 0 and the wavelength of the incident light denoted as *λ*, the grating equation is expressed as follows:

2dsin2i=mλ (3)

### 2.3 Design of zoom lens group

**Fig 5:**
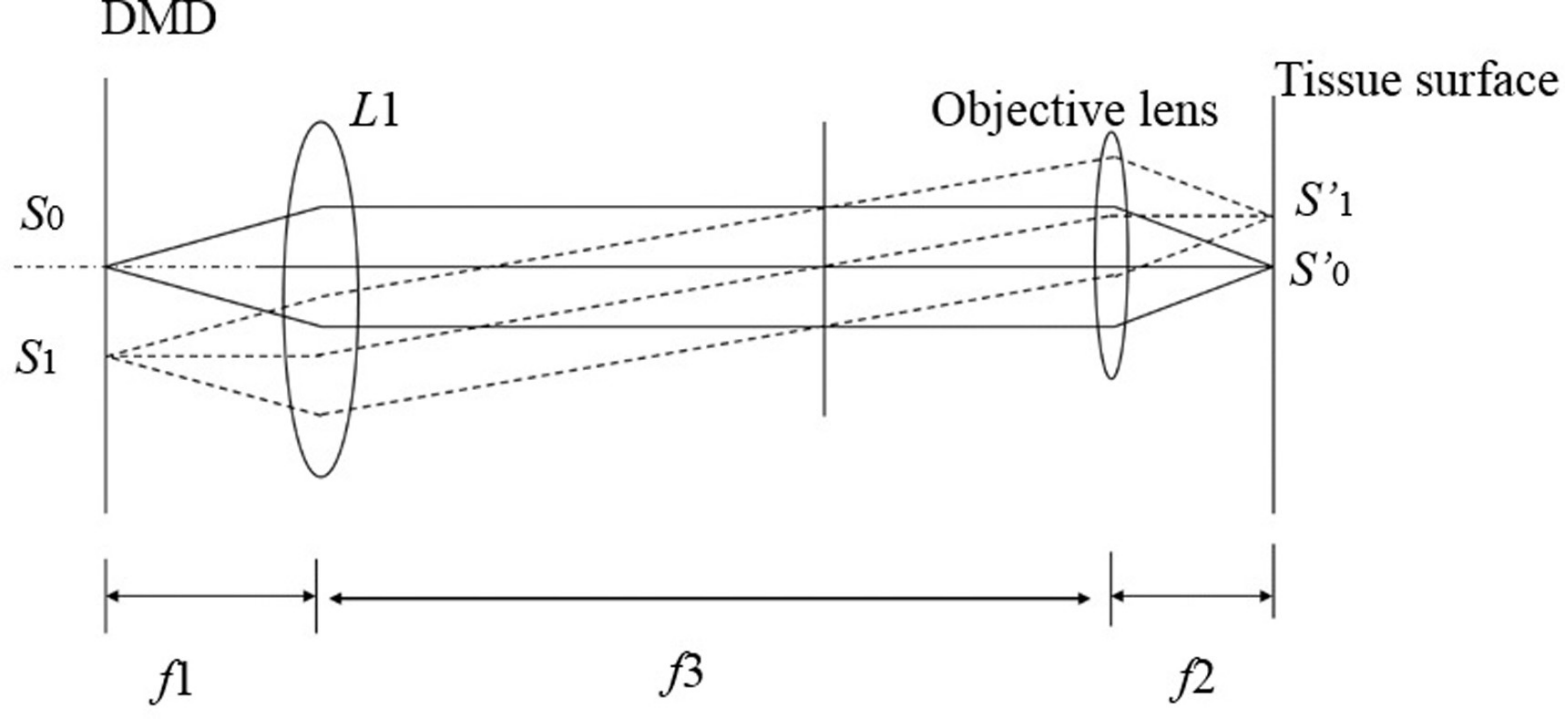
Schematic diagram of DMD compact projection.

After the Digital Micromirror Device (DMD) shapes the laser beam, a suitable optical path is required to scale the shaped spot and achieve a sufficiently small cutting line width, which is crucial for effectively cutting small tissue samples. To maintain the stability of the laser microdissection system, this study employs a telescope system to scale the projected pattern, as illustrated in Fig 5.

In the figure, L1 represents the lens located outside the microscope in Fig 1, which, in conjunction with the microscope objective lens, forms the telescope zoom system. The objective lens in Fig 5 corresponds to the objective lens of the microscope. According to geometric optics theory, a proportional relationship exists between the line width of the etched line and the line width of the DMD mode. The calculation is as follows:

$$W=\frac{ndf_{2}}{f_{1}}$$ (4)

The narrowed laser beam width is denoted by *W*, the number of opened micromirrors is denoted by *n*, and *d* represents the size of the micromirror. Additionally, *f*_1_ represents the equivalent focal length of the system components, and *f*_2_ represents the equivalent focal length of the objective lens. The distance *f*_3_ between the two lenses is equal to the sum of *f*_1_ and *f*_2_. In this system, *f*_1_ = 1000 mm, *f*_2_ = 10 mm, and the magnification obtained according to Eq 1 is 100 times.

### 2.4 Vacuum system design

**Fig 6:**
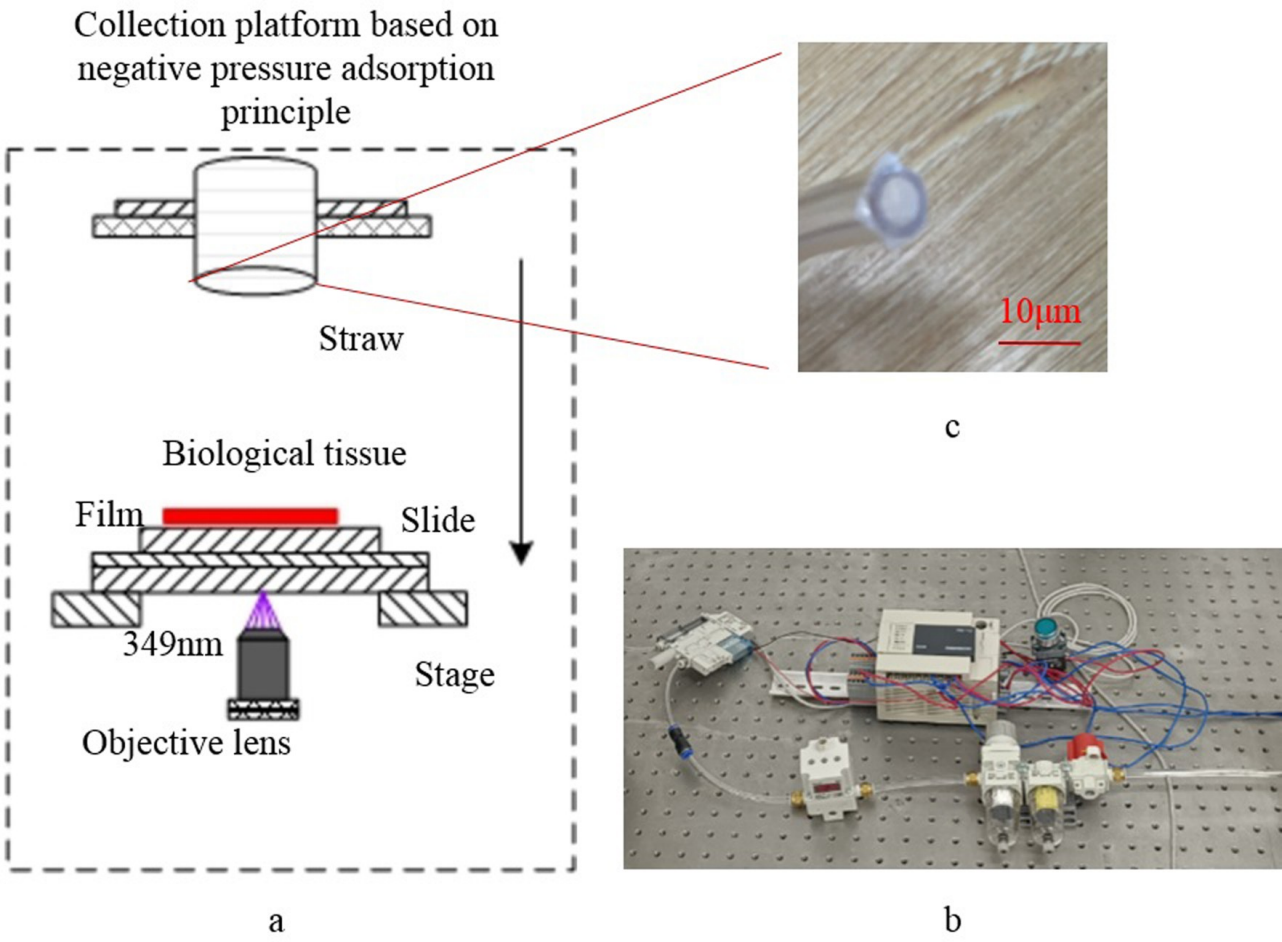
Overall view of the negative pressure adsorption system. (a) Working diagram of the negative pressure adsorption system (b) Physical view of the negative pressure adsorption system (c) Enlarged view of the end of the actuator suction pipe.

**Fig 7:**
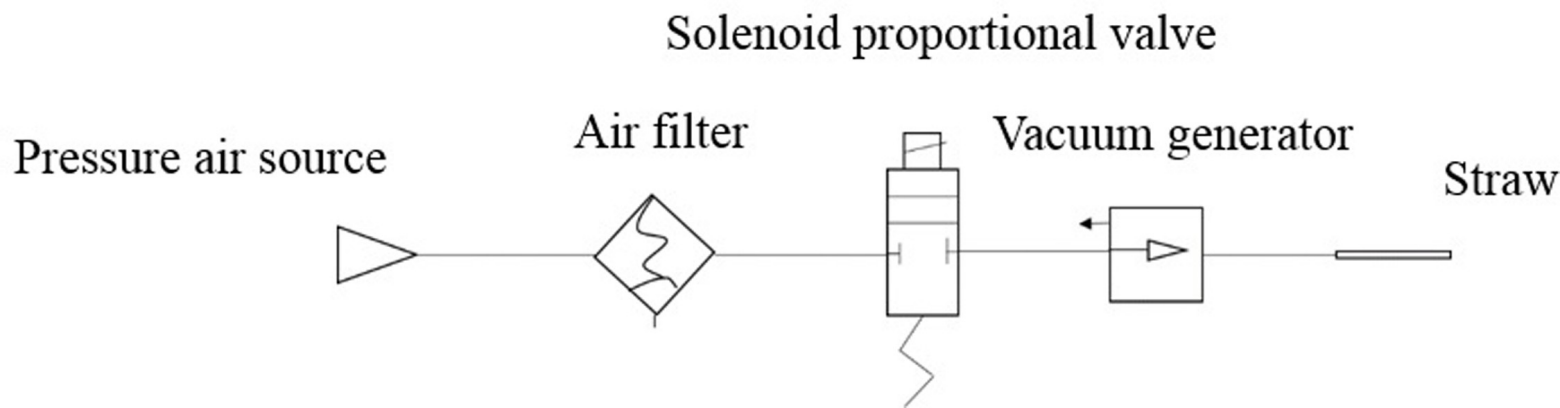
Working principle diagram of the negative pressure adsorption system.

The physical setup and principles of the negative pressure adsorption system used in this study are depicted in Figs 6 and 7.The negative pressure adsorption system comprises a suction pipe, a vacuum generator, a PLC controller, an electromagnetic proportional valve, a filtration system, and a gas source. The inner diameter of the straw employed in the experiment was 4 mm, with its end covered by a PET film layer featuring multiple holes distributed across its surface, facilitating the absorption of target tissue without penetration. The vacuum generator employed was the ZK2G07R5ALA-06 model manufactured by SMC Company. Additionally, the solenoid proportional valve and filter system, also sourced from SMC, were utilized to regulate intake pressure and filter impurities, respectively. The PLC controller used was the FX3U-16MR/ES controller manufactured by Mitsubishi Company, tasked with controlling the vacuum generator’s operational status. The air source was provided by a vacuum compressor.

### 2.5 Experimental sample preparation

In this study, 6-week-old Balb/c mice were used. The experimental protocol involving animal subjects was approved by the Ethics Committee for the Management of Experimental Animals at Huaqiao University School of Medicine. To induce anesthesia, the mice were administered a mixture of ketamine (100 mg/ml) and diazepam hydrochloride (10 mg/ml) at a ratio of 10:1 via intraperitoneal injection while being housed in a well-ventilated cage. Anesthesia was confirmed by the absence of response and slow breathing. Subsequently, euthanasia was performed using cervical dislocation.

To extract brain tissue, the mouse’s head was rinsed with normal saline, and the skin was incised with scissors to expose the skull. The skull was carefully opened with a scalpel to reveal the brain tissue, which was then delicately removed using dissecting forceps. The extracted brain tissue was immediately immersed in 10% neutral buffered formalin for fixation, with a fixation duration of 24 hours at room temperature to ensure complete fixation. Following fixation, the tissue was washed three times with PBS buffer for 5 minutes each. Subsequently, the fixed brain tissue was gradually dehydrated in sequential ethanol solutions (70%, 80%, 90%, and 95% ethanol for 30 minutes each, followed by absolute ethanol for two 30-minute intervals), and then cleared in xylene twice for 1 hour each. The brain tissue was subsequently saturated by overnight immersion in molten paraffin wax, followed by embedding, sectioning into 5-micron thickness slices, and dewaxing. The sections were stained with hematoxylin for 5–10 minutes, rinsed with tap water, and then counterstained with eosin for 1–2 minutes before another rinse with tap water. Finally, the sections were sequentially rehydrated in different ethanol concentrations (70%, 80%, 90%, and 95% ethanol for 5 minutes each, followed by absolute ethanol twice for 5 minutes each) and xylene (two 5-minute soaks), before being sealed with neutral gum.

For immunohistochemical sections, mice were immobilized on an operating table, and the mammary gland area was cleansed with 70% ethanol before the mammary gland tissue was excised. The removed tissue was promptly fixed in 10% neutral buffered formalin for 24 hours at room temperature. Following fixation, the mammary gland tissue underwent gradual dehydration (30 minutes each in 70%, 80%, 90%, and 95% ethanol, followed by two 30-minute intervals in absolute ethanol), embedding, sectioning into 5-micron thickness slices, and dewaxing. Antigen retrieval was then performed in a 95°C water bath using 10 mM phosphate buffer (pH 6.0), followed by protein blocking with 5% bovine serum protein. Subsequently, primary antibodies against rabbit anti-cell markers were applied at appropriate concentrations and incubated overnight at room temperature. Following this, an appropriate amount of HRP-labeled secondary antibody was added and incubated for 1 hour at room temperature. Finally, DAB color reagent and a color-developing substrate were applied. The slices were then gradually dehydrated (1-minute incubations in 70%, 95%, and 100% ethanol), followed by sealing with hyaluronic acid ester.

## 3 Experimental and discussion

### 3.1 Laser microdissection experiment

**Fig 8:**
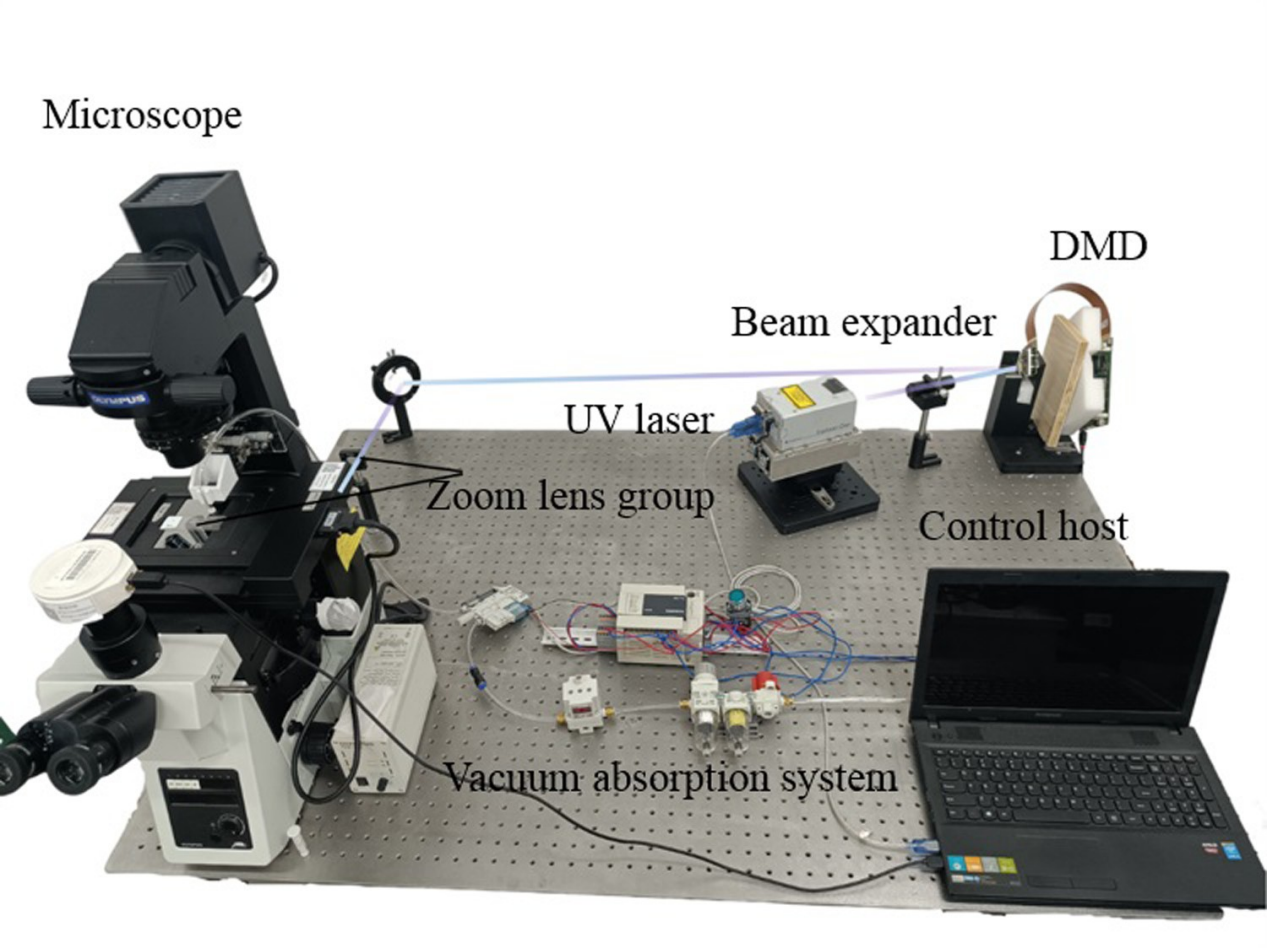
Physical picture of the DMD-based laser microdissection system experimental system.

The experimental device’s actual image is depicted in Fig 8.

**Fig 9:**
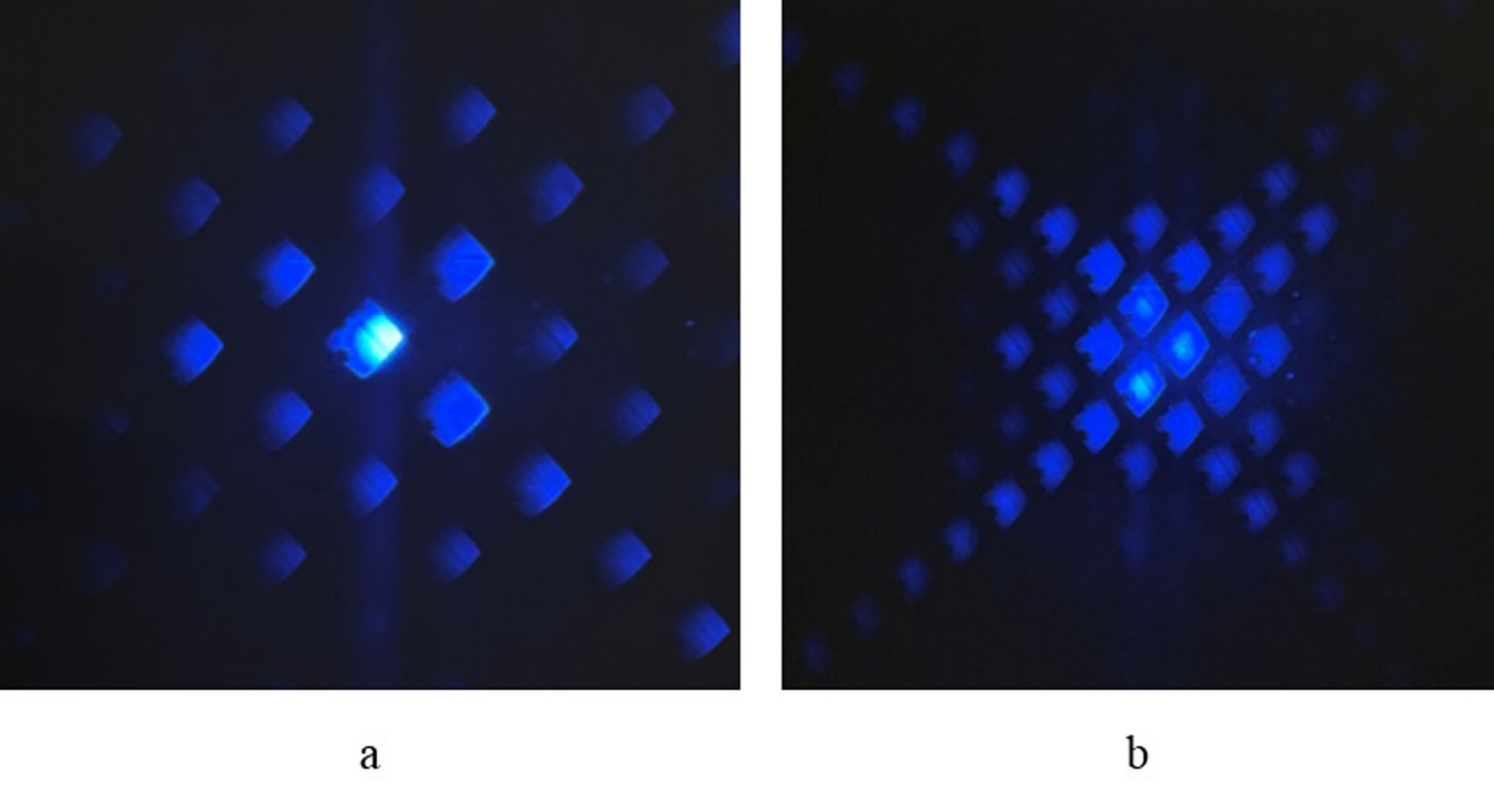
Energy distribution diagram of different diffraction orders. (a) Distribution of laser energy when the incident angle is 24°. (b) Distribution of laser energy at other angles.

Utilizing the DMD blazed grating model, this article utilizes a laser wavelength of 349nm and a DMD micromirror size of 13.68μm. It is apparent that at a system incident angle of 24°, the majority of energy is concentrated on the zero-order diffraction, thereby achieving maximum energy distribution. This is illustrated in Fig 9(A), demonstrating the concentration of energy in the zero-order diffraction at the mentioned incident angle. Conversely, Fig 9(B) depicts the non-uniform distribution of laser energy across various diffraction orders at alternative angles.

**Fig 10:**
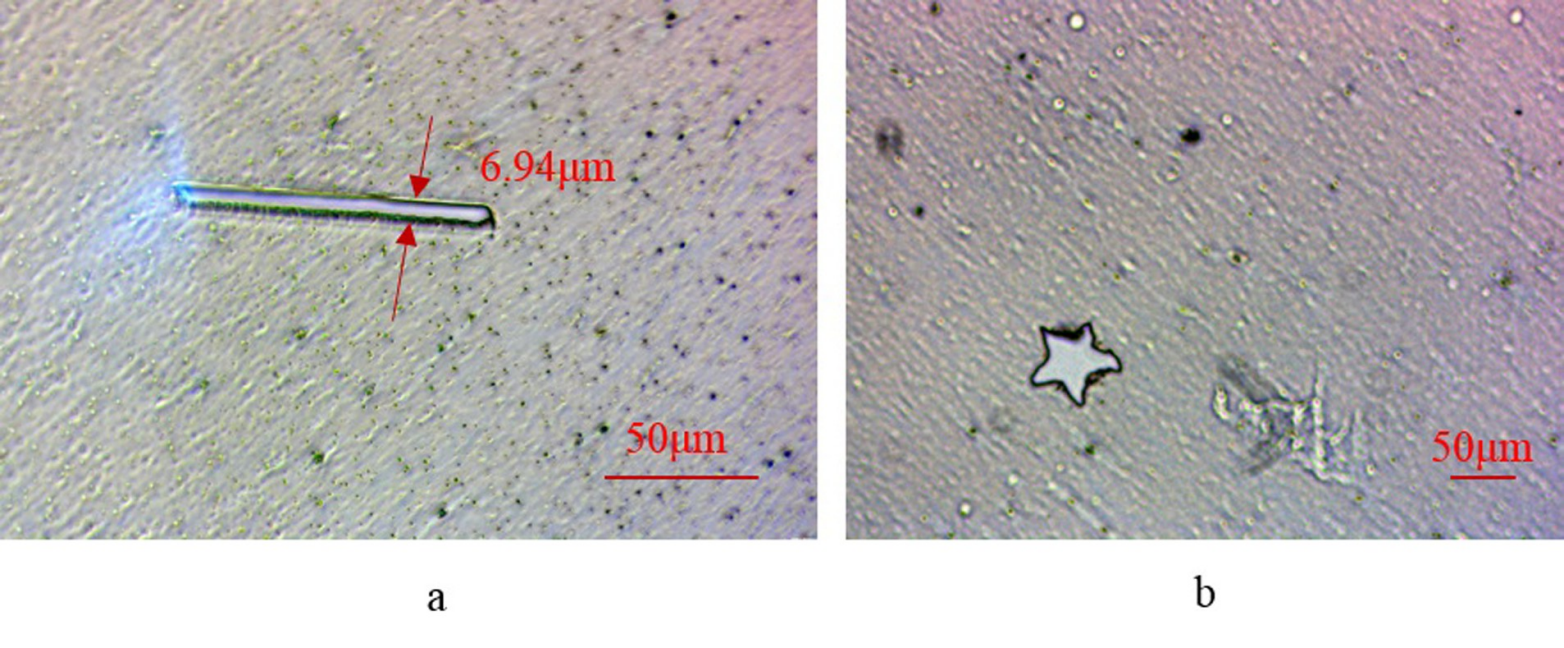
Dual mode cutting on PET film. (a) Point scanning cutting (b) Projection cutting.

**Fig 11:**
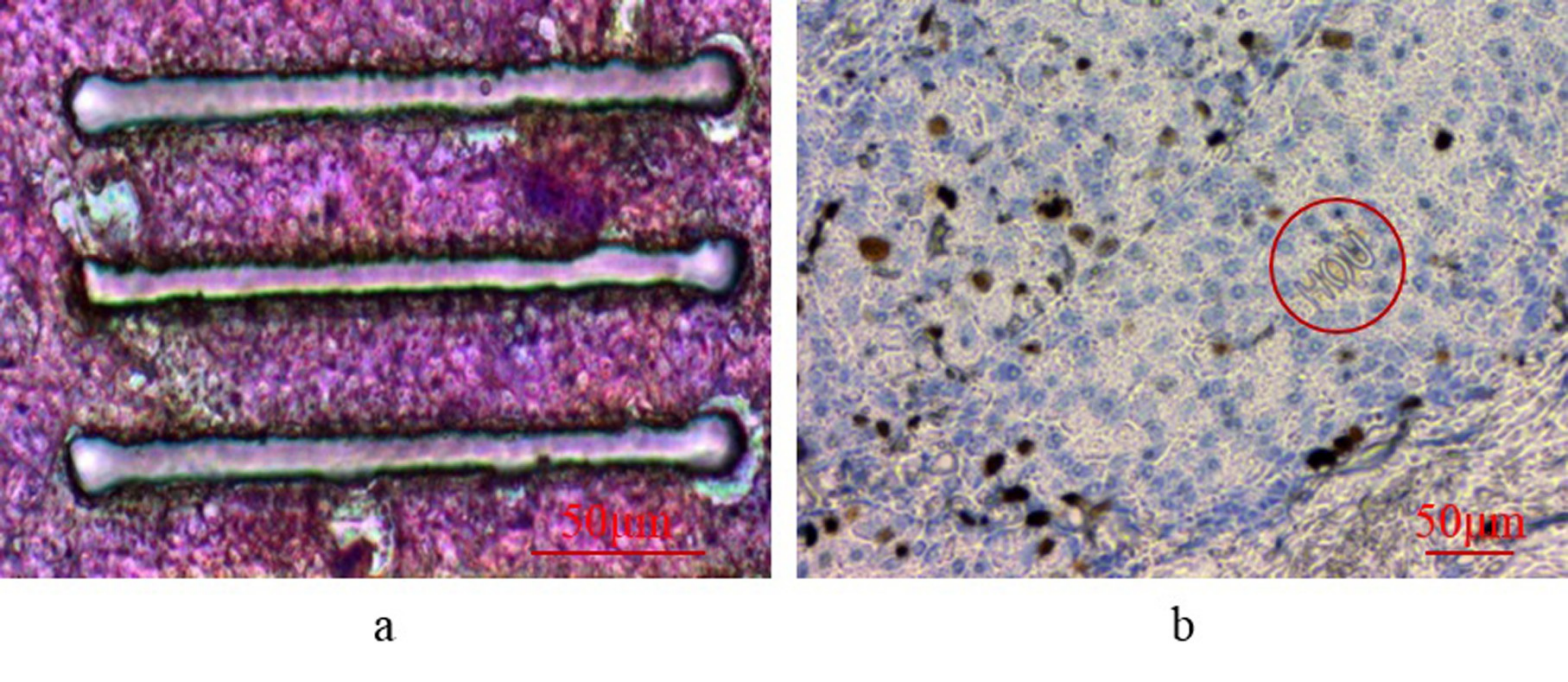
Dual-mode cutting on biological tissue. (a) Point scanning cutting (b) Projection cutting.

To validate the efficacy of the dual-mode cutting improvement system, verification tests were conducted on two materials: PET film and biological tissue. The experimental findings are presented in Figs 10 and 11. Fig 10(A) demonstrates the feasibility of the telescope system in scaling patterns on 1.4 μm thick PET film. With a loaded spot diameter of 50 microns on the DMD, the system’s L1 lens focal length f1 is 1000mm, while the 40x objective lens has an equivalent focal length f2 of 10mm, resulting in a reduction factor of 100x. In the single-factor experiment, a minimum laser current of 3A, a repetition frequency of 1KHz, and a cutting speed of 30mm/s were selected for cutting biological tissue. At these settings, the theoretical line width is calculated to be 6.84 μm, closely matching the actual line width observed in Fig 10(A) (6.94 μm), thus meeting system requirements. Fig 10(B) illustrates the projected cross-section of a five-pointed star using a laser current of 3A and a pulse frequency of 1KHz, with 300 micromirrors spaced between two vertices horizontally. Moving to Fig 11, the results of the cutting experiment on biological tissue are shown. Employing a laser current of 3A, a repetition frequency of 1KHz, and a point scanning cutting speed of 30mm/s, the system operates on 5μm thick biological tissue. In Fig 11(A), the DMD utilizes 100 micromirrors in point cutting mode, while in Fig 11(B), the letters are delineated by a width of 30 micromirrors.

Figs 10 and 11 collectively validate the efficacy of the DMD-based laser microdissection method in both point scanning mode for cutting straight lines and projection cutting mode for one-time projection cutting of anisotropic patterns on PET films and biological tissues. Leveraging the DMD’s laser micro-cutting technique, precise contours can be accurately projected to the intended location, enabling swift and accurate cutting. This method surpasses traditional cutting approaches constrained by mechanical movement, allowing for enhanced cutting precision and the handling of smaller target sizes. Notably, existing laser micro-dissection systems typically feature a minimum cutting target larger than 10 microns [27–29].

In Fig 11(A), noticeable dark burn marks were observed around the incision. This phenomenon primarily results from the Gaussian distribution of energy emitted by the ultraviolet laser used in this platform’s circular laser spot. The higher energy at the center of the spot directly vaporizes tissue to form the incision, whereas the lower energy distribution at the periphery of the circular spot fails to vaporize tissue completely, resulting only in surface burns and the formation of dark burn areas.

To mitigate the occurrence of dark burns from ultraviolet laser cutting in biological tissue, optimizing the energy distribution of the laser spot to achieve uniform energy across the entire circular spot can be effective in preventing localized burning. Additionally, optimizing the process parameters of laser microsurgery can help minimize the burn area as much as possible.

**Fig 12:**
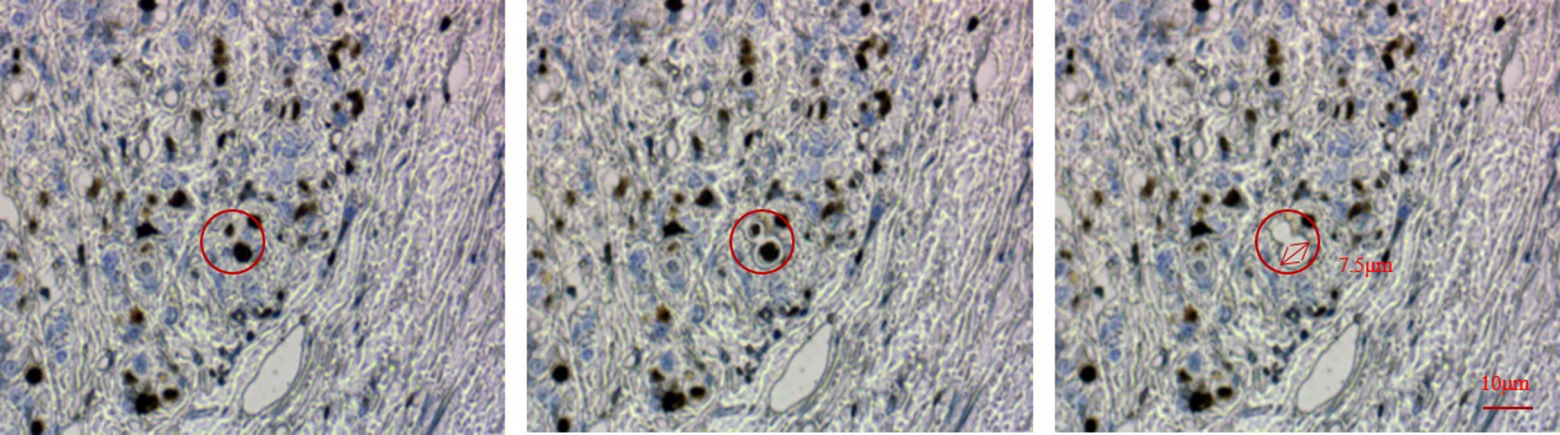
Projection cutting process and results.

To further scrutinize the system’s cutting accuracy, the experiment utilized a 40x objective lens offering 100x magnification. A DMD with a side length of 13.68 μm was employed to generate a circular cutting pattern with a line width equivalent to 20 micromirror lengths, ensuring precise targeting. The cutting laser operated at a current of 3A with a pulse frequency of 1KHz. The experiment utilized 5-micron thick immunohistochemical sections of mouse breast tissue as samples. Fig 12 illustrates the cutting process alongside its corresponding outcomes.

The experimental results showcase the capability of the DMD projection cutting mode to achieve smaller cutting sizes, with diameters measuring up to 7.5μm, surpassing those achievable with existing laser microdissection systems. This technological advancement facilitates the swift and precise isolation of minute biological tissues.

### 3.2 Cutting target capture experiment

**Fig 13:**
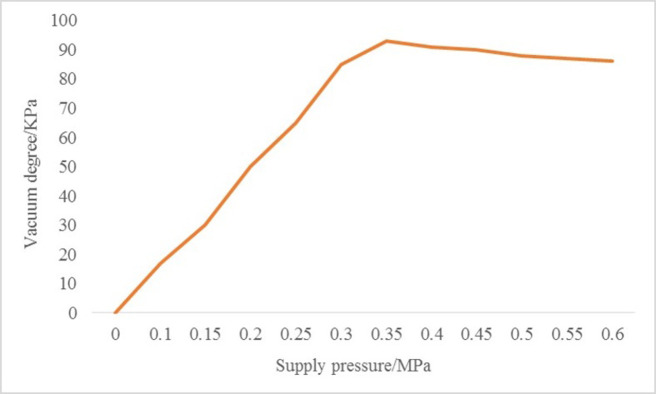
Exhaust characteristic diagram.

**Fig 14:**
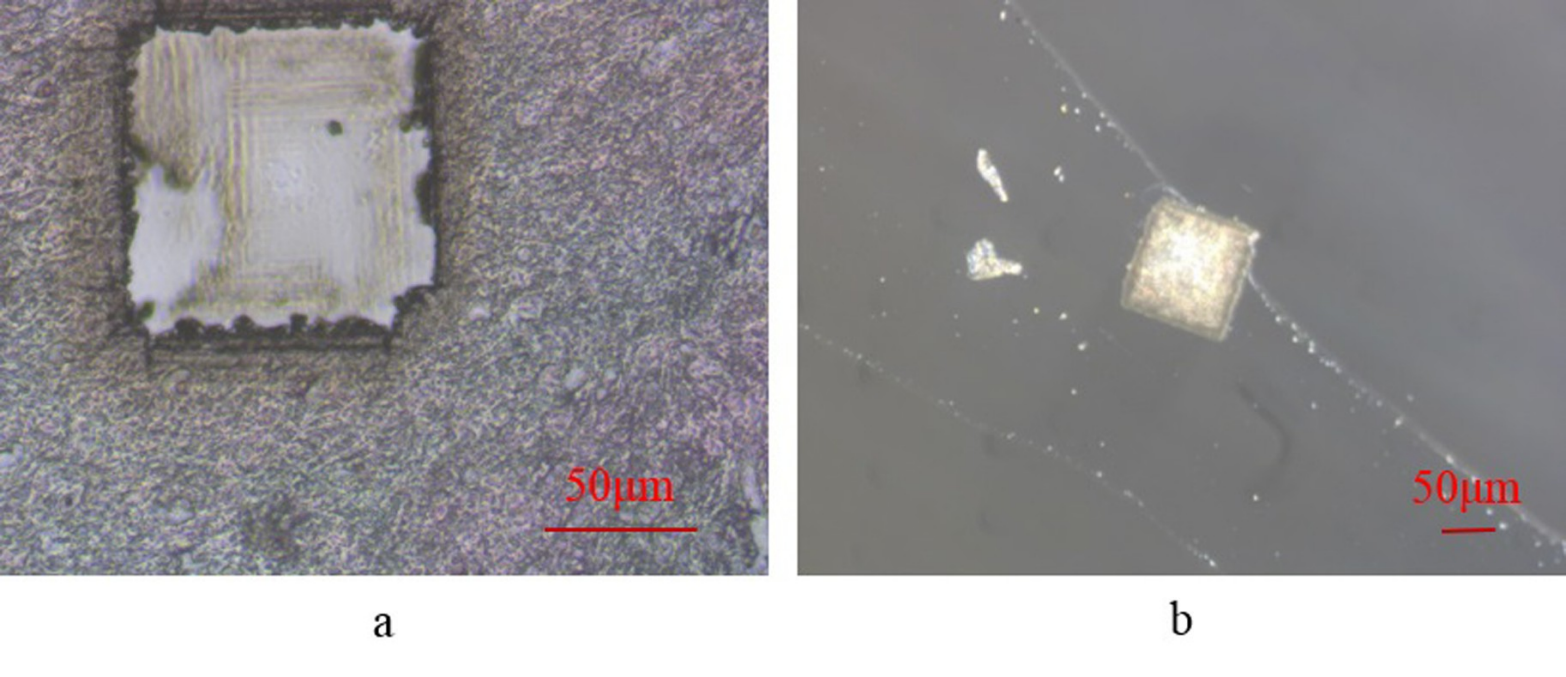
The capture process of the negative pressure adsorption system. (a) the remaining tissue portion after the target tissue is captured. (b) the target tissue released on the slide after being captured.

To efficiently transfer adsorbent substances into the designated container, the vacuum suction of the micro pipette should not be excessively strong. As long as it meets the minimum suction requirement, it suffices to fulfill the task. The pressure characteristic curve of the vacuum generator (refer to Fig 13) illustrates how the vacuum level fluctuates with changes in the supply pressure. In this investigation, we identified the optimal operating conditions for the vacuum generator, setting the supply pressure at 0.45 MPa. For this experiment, the adsorption film featured a pore size of 5 micrometers. Utilizing HE-stained 5-micrometer mouse brain tissue slices as samples, each side measuring 100 micrometers square, we conducted the experiment. Fig 14(A) displays the remaining tissue portion post-target tissue capture, while Fig 14(B) exhibits the captured target tissue released onto a glass slide.

To assess the capture efficiency of the negative pressure adsorption system, we conducted experiments to determine capture rates across various target volumes. Traditional targets typically range from 10 μm to 100 μm. Therefore, for our experiments, we employed sample sizes of 30 μm, 60 μm, and 90 μm. The air compressor’s supply pressure was maintained at 0.45 MPa. Utilizing HE-stained 5 μm sections of mouse brain tissue as samples, we conducted multiple tests for each target size, repeating the process 50 times. The resulting capture rates were 98%, 94%, and 92%, respectively. As the sample area increases, the contact area between the sample and the slide’s film also expands. This increased contact leads to heightened adhesion, making sample capture more challenging and consequently decreasing the capture rate.

**Fig 15:**
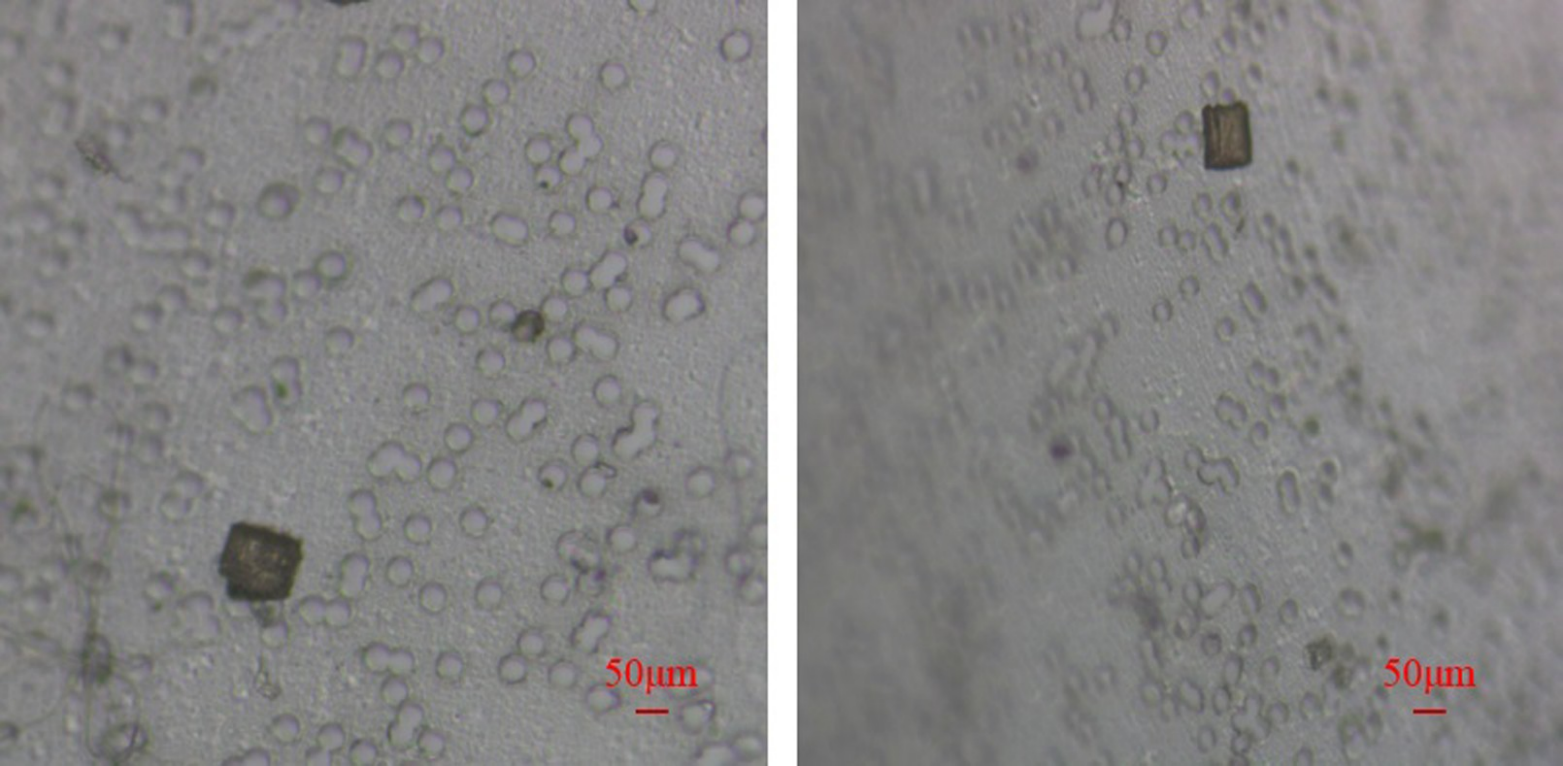
Capture multiple target tissues distributed in different areas on the capture device and captured simultaneously.

Due to the extensive surface area of the membrane at the target collection end, multiple targets can be captured simultaneously in a single operation without necessitating multiple movements. We verified the system’s capability to capture multiple targets simultaneously. Fig 15 illustrates the tissue conditions of HE-stained sections, each with a side length of 100 μm and a thickness of 5 μm from mouse brain tissue, distributed across various areas of the capture device and simultaneously captured by the system.

## 4. Conclusions

This paper introduces a laser microdissection system integrating DMD for spatial light modulation, featuring dual cutting modes and a negative pressure adsorption collection method. By controlling the flip of the DMD micromirror element during cutting, the device offers two cutting modes. The one-time projection cutting mode enables swift cutting of shaped targets smaller than 10 μm. The negative pressure adsorption system exhibits capture rates of 98%, 94%, and 92% for three sizes of mouse brain tissue targets (30 μm, 60 μm, and 90 μm), respectively. Additionally, simultaneous capture of multiple targets was successfully demonstrated. Overall, these results suggest that our newly devised dissection device enhances dissection accuracy and effectiveness. The tissues and cells collected using this system hold significant potential for various downstream applications.

## Supporting information

S1 Fig Specific data of the experimental platform. (PDF)

S2 Fig Experiment on the capture rate of targets of different sizes. Targets with a 30μm edge length captured under a 10x objective. Targets with a 60μm edge length captured under a 10x objective. Targets with a 90μm edge length captured under a 10x objective.
(PDF)

S1 Table Raw data of the capture success rate experiment. (DOCX)

S2 Table Vacuum generator exhaust characteristics. (DOCX)
