# Supplementary figures and images for: Laser microdissection system based on structured light modulation dual cutting mode and negative pressure adsorption collection
Source: PLoS One. 2024 Aug 26;19(8):e0308662. doi: 10.1371/journal.pone.0308662 (PMC11346911; doi:10.1371/journal.pone.0308662)

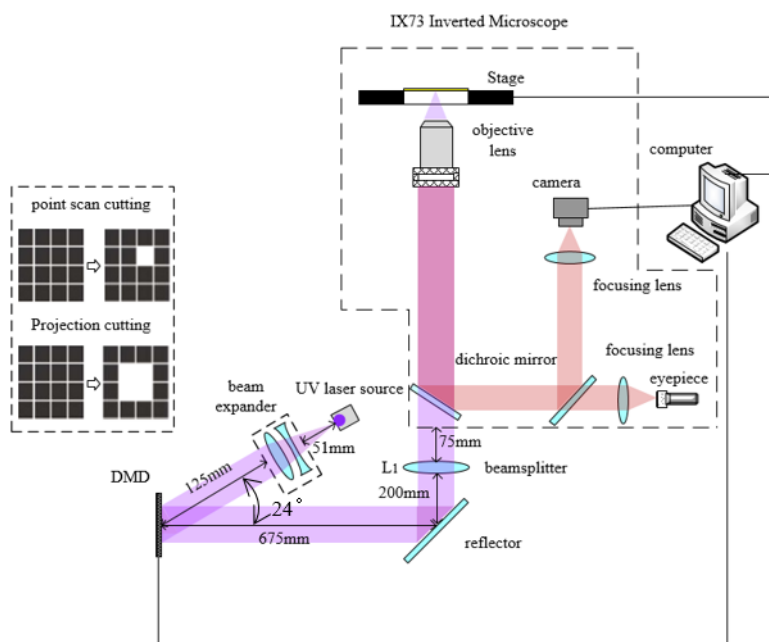

S1\_Fig. Specific Data of the Experimental Platform

Supplement: S1 Fig — (PDF) [file pone.0308662.s001.pdf]
