# Supplementary material for: Laser microdissection system based on structured light modulation dual cutting mode and negative pressure adsorption collection
Source: PLoS One. 2024 Aug 26;19(8):e0308662. doi: 10.1371/journal.pone.0308662 (PMC11346911; doi:10.1371/journal.pone.0308662)
Supplement: S2 Fig — Targets with a 30μm edge length captured under a 10x objective. Targets with a 60μm edge length captured under a 10x objective. Targets with a 90μm edge length captured under a 10x objective. (PDF) [file pone.0308662.s002.pdf]

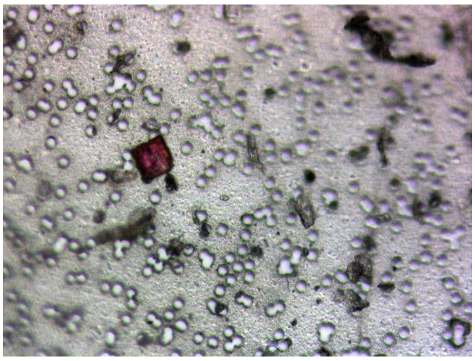

a

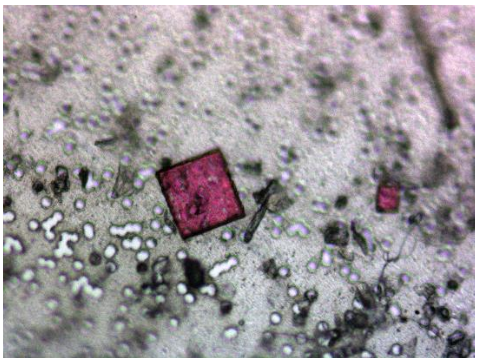

b

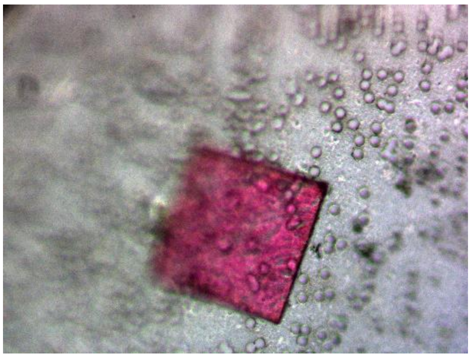

c

S2_Fig. Experiment on the Capture Rate of Targets of Different Sizes

(a) Targets with a 30μm edge length captured under a 10x objective.(b)Targets with a 60μm edge length captured under a 10x objective.(c) Targets with a 90μm edge length captured under a 10x objective
