# Supplementary material for: Laser microdissection system based on structured light modulation dual cutting mode and negative pressure adsorption collection
Source: PLoS One. 2024 Aug 26;19(8):e0308662. doi: 10.1371/journal.pone.0308662 (PMC11346911; doi:10.1371/journal.pone.0308662)
Supplement: S1 Table — (DOCX) [file pone.0308662.s003.docx]

S3_Table. Raw Data of the Capture Success Rate Experiment

| Serial Number | 30μm | 60μm | 90μm |
| --- | --- | --- | --- |
| 1 | √ | √ | √ |
| 2 | √ | √ | √ |
| 3 | √ | √ | √ |
| 4 | √ | √ | √ |
| 5 | √ | √ | √ |
| 6 | √ | √ | √ |
| 7 | √ | √ | √ |
| 8 | √ | √ | √ |
| 9 | √ | √ | √ |
| 10 | √ | √ | √ |
| 11 | √ | × | √ |
| 12 | √ | × | √ |
| 13 | √ | √ | √ |
| 14 | √ | √ | √ |
| 15 | × | √ | √ |
| 16 | √ | √ | √ |
| 17 | √ | √ | × |
| 18 | √ | √ | √ |
| 19 | √ | √ | √ |
| 20 | √ | √ | √ |
| 21 | √ | √ | √ |
| 22 | √ | √ | √ |
| 23 | √ | √ | √ |
| 24 | √ | √ | √ |
| 25 | √ | √ | √ |
| 26 | √ | √ | √ |
| 27 | √ | × | √ |
| 28 | √ | √ | × |
| 29 | √ | √ | √ |
| 30 | √ | √ | √ |
| 31 | √ | √ | √ |
| 32 | √ | √ | √ |
| 33 | √ | √ | √ |
| 34 | √ | √ | √ |
| 35 | √ | √ | √ |
| 36 | √ | √ | √ |
| 37 | √ | √ | √ |
| 38 | √ | √ | × |
| 39 | √ | √ | √ |
| 40 | √ | √ | √ |
| 41 | √ | √ | √ |
| 42 | √ | √ | √ |
| 43 | √ | √ | × |
| 44 | √ | √ | √ |
| 45 | √ | √ | √ |
| 46 | √ | √ | √ |
| 47 | √ | √ | √ |
| 48 | √ | √ | √ |
| 49 | √ | √ | √ |
| 50 | √ | √ | √ |
