# Supplementary material for: Laser microdissection system based on structured light modulation dual cutting mode and negative pressure adsorption collection
Source: PLoS One. 2024 Aug 26;19(8):e0308662. doi: 10.1371/journal.pone.0308662 (PMC11346911; doi:10.1371/journal.pone.0308662)
Supplement: S2 Table — (DOCX) [file pone.0308662.s004.docx]

S4_Table. Vacuum generator Exhaust Characteristics

| Supply pressure /MPa | 0.1 | 0,15 | 0,2 | 0,25 | 0,3 | 0.35 | 0.4 | 0.45 | 0.5 | 0.55 | 0.6 |
| --- | --- | --- | --- | --- | --- | --- | --- | --- | --- | --- | --- |
| Vacuum pressure /kPa | -15 | -30 | -50 | -65 | -85 | -93 | -91 | -90 | -88 | -87 | -86 |
